# Syndrome coronarien aigu en post-partum secondaire à une dissection coronaire spontanée: à propos d'un cas

**DOI:** 10.11604/pamj.2015.20.249.6410

**Published:** 2015-03-16

**Authors:** Nabil El Malki Berrada, Achraf Zaimi, Abdellatif Ezzouak, Hassan Belhachmi, Alaeeddine Lagziri, Sara Ratbi, Badre eddine Elyounassi

**Affiliations:** 1 Service de cardiologie, Hôpital Militaire Moulay Ismail, Meknès, Morocco

**Keywords:** IDM, post partum, dissection coronaire, Myocardial infarction, post partum, cooronary dissection

## Abstract

L'IDM per gravidique est une complication grave qui entraine une morbidité et une mortalité maternelle élevée. Bien que l'athérosclérose soit la cause d'IDM la plus fréquente dans la population générale, elle n'est observée que chez un tiers des femmes enceintes. Chez ces dernières, la cause la plus fréquente d'IDM était la dissection coronaire. Nous rapportons l'observation clinique d'une jeune femme de 24 ans, sans facteur de risque cardiovasculaire, qui a présenté un IDM antérieur étendu 15 jours après un accouchement, en rapport avec une dissection de la partie proximale de l'artère inter ventriculaire antérieure. Le traitement de cette pathologie n'est pas consensuel, et peut faire appel, selon la présentation clinique et angiographique, au traitement médical, à une revascularisation par pontage aorto-coronaire avec une résection de l'hématome de la paroi artérielle, ou à l'angioplastie transluminale. Le pronostic semble assez favorable quoique controversé.

## Introduction

La dissection spontanée d´une artère coronaire (DSAC) est une cause rare de syndrome coronarien aigue (< 4%) [1], avec des mécanismes physiopathologiques mal connus. Elle a été décrite pour la première fois en 1931, au cours d´une autopsie chez une femme de 42 ans décédée après avoir présenter une douleur thoracique. Environ 400 cas documentés de DSAC seulement qui ont été rapportés dans la littérature. Ce qui est probablement sous-estimé en raison d´un nombre important de cas se présentant dans un tableau de mort subite [2]. Nous rapportons l´observation d´un infarctus antérieur du post-partum chez une jeune femme, secondaire à une dissection de l'artère interventriculaire antérieure.

## Patient et observation

**Figure 1:**
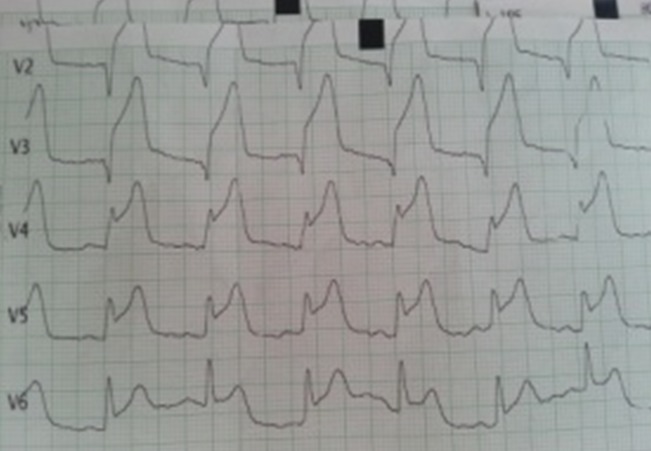
ECG montrant le sus décalage ST au niveau du territoire antérieur

**Figure 2:**
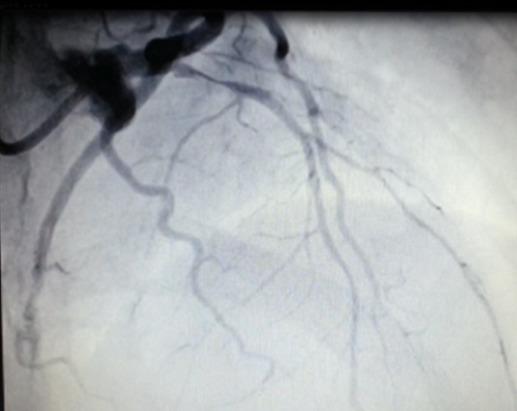
Image coronarographique: dissection de la partie proximale de l'IVA

**Figure 3:**
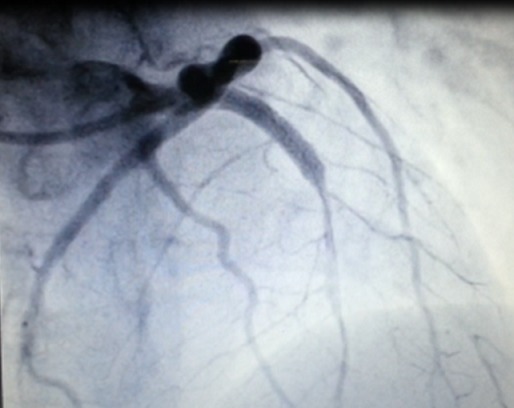
Image coronarographique: angioplastie et mise en place d'un stent sur l'IVA proximale

Il s'agit d'une patiente âgée de 24 ans, sans antécédent pathologique notable ni aucun facteur de risque cardiovasculaire, G2P2, admise à 15 jours du postpartum pour une douleur thoracique aigue rétro sternale constrictive intense, au repos, irradiant vers le bras gauche et la mâchoire inférieure et qui a duré plus de 30 min. L’électrocardiogramme à son admission a objectivé un sus décalage du segment ST au niveau du territoire antérieur (Figure 1). Pour évaluer sa fonction cardiaque, une échocardiographie a été réalisé au lit de la patiente et qui a montré une hypokinésie sévère de la paroi antéro-septale et de la pointe avec une fraction d’éjection estimée à 45 - 50%. La patiente a reçu immediatement une dose de charge du clopidogrel (4cp de 75mg) + l'enoxaparine 0.6 ml en sous cutané + aspirine 250mg en IVD et fut adressée directement à la salle de cathétérisme. La procédure angiographique a mis en évidence une dissection coronaire intéressant la partie proximale de l'artère inter ventriculaire antérieure (Figure 2) et indication d'une angioplastie avec mise en place d'un stent (Figure 3). La patiente a bien évolué et elle est sortie sous traitement associant du Clopidogrel 75mg 1cp/j + Aspirine 75mg 1s/j + Bisoprolol 5mg 1cp/j + Simvastatine 20mg 1cp/j.

## Discussion

L'IDM per gravidique est une complication grave qui entraine une morbidité et une mortalité maternelle élevée estimée à 7.3% [3]. Bien que la probabilité de faire un infarctus pendant la grossesse soit très faible, estimée à 1 sur 16 000 accouchements, le risque est toujours trois à quatre fois supérieur chez les femmes enceintes comparées aux femmes du même âge qui n´attendent pas d´enfant, Et plus les femmes retardent leur première grossesse, plus l´incidence des IDM devrait augmenter. L´athérosclérose est la cause d´IDM la plus fréquente dans la population générale. Néanmoins, elle n´est observée que chez un tiers des femmes enceintes. Chez ces dernières, la cause la plus fréquente d´IDM est la dissection coronaire. Cette dernière se définit par une séparation au sein de la paroi artérielle coronaire secondaire à une hémorragie intra murale, avec ou sans déchirure de l'intima, créant un faux chenal. Cette séparation peut siéger entre l'intima et la média ou entre la média et l'adventice. Angiographiquement, il s'agit d'une image de flux dans deux lumières (séparées par une zone non opacifiée). Sa physiopathologie reste hypothétique, elle est souvent associée à des anomalies artérielles prédisposantes, avec ou sans facteur de stress précipitant de nature émotionnelle, physique ou hormonale (Tableau 1). La dissection coronaire du peripartum est liée aux changements hormonaux, à l'augmentation du débit sanguin ainsi qu'aux changements de l'architecture de la paroi artérielle avec incrimination de l´effort physique intense produit lors du travail et de l´accouchement. Un diagnostic précoce est crucial pour une prise en charge adéquate de la DSAC. Cependant, L'angiographie reste un examen indispensable malgré ses limites à différencier entre l'origine athérosclérotique et non athérosclérotique des obstructions coronaires. Le traitement de cette pathologie n´est pas consensuel, et peut faire appel, selon la présentation clinique et angiographique, au traitement médical, à une revascularisation par pontage aorto-coronaire avec une résection de l´hématome de la paroi artérielle, ou à l´angioplastie transluminale. Le traitement médical comporte une bithérapie d'antiagrégants plaquettaires (aspirine + clopidogrel), considérant qu'une grande proportion de la DSAC implique une déchirure intimale, formant ainsi un milieu prothrombotique. On considère que la réduction du thrombus au niveau du faux chenal par des agents AAP pourrait théoriquement diminuer la compression sur la vraie lumière. Le rôle du traitement anticoagulant est controversé vu le risque de l'extension de la dissection. Ce risque est compensé par le bénéfice potentiel de la résolution du thrombus recouvrant et l'amélioration de la perméabilité artérielle. Les thrombolytiques devraient être évité dans la DSAC vu le risque accru d'une extension de la dissection. Dans une étude rétrospective de Shamboo et al [4], 60% des patients thrombolysés ont nécessité le recours à une intervention coronaire percutanée ou une chirurgie de sauvetage.

**Tableau 1:** Facteurs étiologiques de la DSAC

Artériopathies présdisposantes	Facteurs de stress prédisposants
Athérosclérose	Exercice intense
Grossesse: postpartum ou antepartum	Stress émotionnel intense
Connectivite: dysplasie fibromusculaire, syndrome De Marfan, Nécrose médiale kystique, Ehler Danlos	Travail
Maladie de système: LEAD, maladie de crohn, sarcoïdose, PAN	Cocaïne
Spasme des artères coronaires	-

En extrapolant les bénéfices des B-bloquants dans la dissection aortique, ils sont administrés dès la phase aigue de la DSAC ainsi qu’à long terme. Les dérives nitrés sont utiles à la phase aigue pour soulager les symptômes secondaires au vasospasme, mais ne sont pas couramment utilisés à long terme. L'administration des statines pour des lésions non athérosclérotiques n'a pas été encore étudié. On les préconise ces les patients dyslipidémiques. L´angioplastie avec pose de stent a montré une efficacité de 91% dans la série de 32 patients de Moukarbel et Alam, mais avec un risque majeur de cathétérisation du faux chenal, voire stenting de ce dernier. Généralement un traitement médical avec une intervention coronarienne percutanée (ICP) sont suffisants pour rétablir la circulation coronaire ainsi qu'une hémodynamique stable [5]. La chirurgie par pontage aorto-coronaire est indiqué dans la dissection du tronc commun coronaire gauche, et dans les dissections impliquant plusieurs coronaires. Dans une série de 23 cas, Vanzetto et al. [6] ont rapporté que le pontage aorto-coronaire a été réalisée chez cinq patients, l'angioplastie chez 8 patients, tandis que 10 patients ont été mis sous traitement médical. Le pronostic des patients avec DSAC n´a pas été bien étudié, vu les cas limités rapportés dans les séries publiées. Toutefois, des séries rétrospectives récentes montrent que la plupart des patients survivaient à leur hospitalisation initiale, avec un faible taux de mortalité allant de 0 à 4%. [6–8] La récurrence de la dissection coronaire varie selon les différentes études. Dans une cohorte réalisée par SAW J., le taux de dissection récurrente est d'environ 10% [9]. Pour Tweet et al, ce taux était de 17% (15/87) à 47 mois de suivi, dont 12/15 dissections récurrentes avaient intéressé une artère coronaire différente. Ils ont rapporté un taux d´événements indésirables majeurs après 10 ans estimé à 47% [10]. Dans l´étude de Koller et al, le taux de dissection récurrente au-delà de 24 heures chez les femmes en post-partum était de 17% [11].

## Conclusion

La dissection coronaire est une cause rare mais grave du syndrome coronarien aigu associée à la grossesse dans la majorité des cas. Elle doit être suspectée chez les jeunes femmes multipares présentant une douleur thoracique dans la période périnatale, même en l´absence de facteurs de risque cardiovasculaires. C'est un diagnostic urgent qui se fait grâce à la coronarographie, et le traitement doit être adapté selon les circonstances individuelles. L’évolution à long terme des patientes qui survivent à leur événement de DSAC est généralement bonne. Cependant, elles sont exposées à un risque de dissection récurrente et d’événements cardiovasculaires majeurs. Par conséquent, elles doivent étroitement être surveillées par leur cardiologue.
